# The moderating effect of appearance on the impact of performance rankings in the live streaming market

**DOI:** 10.3389/fpsyg.2022.1011787

**Published:** 2022-11-22

**Authors:** Yasheng Chen, Xian Huang, Sijia Zhao

**Affiliations:** ^1^Department of Accounting, School of Management, Xiamen University, Xiamen, Fujian, China; ^2^Ernst & Young (China) Hua Ming LLP Hangzhou Branch, Hangzhou, China

**Keywords:** relative performance information, broadcaster effort, viewer participation, live streaming performance, beauty premium

## Abstract

The live streaming market is becoming increasingly competitive, and relative performance information regarding broadcasters is available to all participants in the live streaming industry, exacerbating the level of competition. Using data from 42,166 live streams by 293 broadcasters, we investigated two benefits of relative performance information in relation to the live streaming business and how these effects vary when the broadcaster appears competent, trustworthy, likable, or attractive. On the basis of economic and social comparison theory, as well as insights from the herd behavior and beauty premium literature, we predicted and found that relative performance information can improve live streaming performance by either increasing broadcaster effort (the effort-eliciting effect) or encouraging viewer participation (the informational effect), with these effects being stronger when broadcasters look more competent, trustworthy, likable, or attractive. The findings of this study contribute to the live streaming literature by demonstrating that providing relative performance information in the live streaming business can yield both effort-eliciting and informational benefits.

## Introduction

The advent of the 5G era has provided an unprecedented opportunity for the development of the live streaming business. Furthermore, the COVID-19 pandemic has contributed to the rapid rise of the “stay-at-home economy,” which has significantly boosted live streaming consumption (Ingilizian, 2020). Given these trends, live streaming businesses are expanding at a rapid rate. In China, there are more than 200 live streaming apps (Liu et al., 2022), and by December 2021, the number of live streaming users had reached 703 million, accounting for 68.2% of all Internet users (CNNIC, 2022). Various industries are exploring and establishing live streaming business models, and viewers have embraced live streaming for various purposes, including pan-entertainment (Chen and Xiong, 2019; Lin et al., 2021), e-sports (Liu et al., 2022), e-tourism (Xie et al., 2022), and e-commerce (Lu and Chen, 2021; Lo et al., 2022).

The quintessential characteristic of a live stream is that a broadcaster generates and delivers real-time content to an audience, who consumes the content by joining the virtual showroom hosted by the broadcaster. In the process of content consumption, viewers interact with the broadcaster in two distinct ways, which in turn shape the content creation process (Lin et al., 2021; Chen and Liao, 2022). One type of interaction is non-monetary in nature, such as texting with the broadcaster or other viewers, and sending “likes” to the broadcaster (Zhou et al., 2019; Lin et al., 2021). The other type of interaction is tipping, whereby viewers give the broadcaster virtual tokens or gifts that they have purchased from the platform using real money (Lin et al., 2021; Guan et al., 2022; Wu et al., 2022). Viewers can decide when and how much to tip the broadcaster at any time during a live stream, and often engage in both monetary and non-monetary interactions multiple times while watching a live stream (Lin et al., 2021).

The prospects for the live streaming industry have attracted the interest of researchers, and because the broadcaster and viewers are the central elements of a live stream, a growing number of studies have focused on the behavior and characteristics of the participants (Wongkitrungrueng et al., 2020; Li et al., 2021; Lin et al., 2021; Liu and Liu, 2021; Chen and Liao, 2022; Guan et al., 2022; Wu et al., 2022; Xu et al., 2022). In this study, we examine the effects of relative performance information (RPI) on live streaming performance, which is measured by the gifting amount (i.e., viewer tips) that a broadcaster receives on a given day (Chen and Xiong, 2019; Zhou et al., 2019; Lin et al., 2021). RPI refers to information that enables comparisons of a broadcaster’s performance with those of their competitors on a given day. Although live streaming performance measures also include non-monetary factors (Lin et al., 2021), we focus on viewer tips because virtual gifts are the major source of revenue for both broadcasters and live streaming companies (Xiang, 2016; Chen and Xiong, 2019; Zhou et al., 2019). Our focus on RPI is also motivated by the fact that RPI is highly visible in the live streaming business. All participants in the live streaming industry can readily access and compare information on broadcaster performance through third-party or live streaming platforms.

The public accessibility of RPI impacts the behavior of both broadcasters and viewers. From the broadcaster’s perspective, RPI enables broadcasters to know their performance ranking in the market, thereby encouraging social comparisons and driving them to raise their effort level to improve their live streaming performance (i.e., the effort-eliciting effect; Tafkov, 2013). For example, a broadcaster with a relatively poor performance ranking might extend their next live stream in an effort to increase their popularity. From the viewer’s perspective, RPI enables the viewer to understand the preferences of the viewing audience (Lu et al., 2021). Viewers might align their decisions with those of other viewers as a result of the information asymmetry that exists between broadcasters and viewers, resulting in a herding effect that eventually improves the live streaming performance (i.e., the informational effect; Ding and Li, 2019). More precisely, a broadcaster with a higher level of RPI will attract more viewers’ attention and participation than one with a lower level of RPI, thereby increasing their popularity and likelihood of receiving tips from viewers. We also examine the moderating role of the broadcaster’s appearance on the relationship between RPI and live streaming performance. A positive relationship has been found between appearance and wages in various fields (Hamermesh and Biddle, 1994), and that this effect is more pronounced in jobs that demand interpersonal interaction (Stinebrickner et al., 2019). We expect this phenomenon to be evident in the live streaming industry because a live stream involves considerable visual and verbal interaction between broadcasters and viewers. In summary, we posit that the effort-eliciting and informational effects of RPI on live streaming performance will be stronger among broadcasters who appear more competent, trustworthy, likable, or attractive (Graham et al., 2017).

To test our predictions, we collected data for 42,166 live streams by 293 broadcasters on YY Live, a leading online streaming entertainment services provider in China (Chen and Xiong, 2019), from 1 May 2020 to 31 October 2020. In addition, six Generation Z (Gen Z) raters were recruited to evaluate the broadcasters’ appearance. We considered Gen Z raters to be appropriate for evaluating the broadcasters’ appearance because they are the primary consumers of live streams and interact with broadcasters during live streams (QuestMobile, 2019). After being provided with standardized screenshots of the broadcasters, each rater independently evaluated each broadcaster’s appearance in four dimensions: competence, trustworthiness, likeability, and attractiveness. The results showed that RPI can improve live streaming performance by either increasing the broadcaster’s effort or encouraging viewer participation, with these effects being stronger when broadcasters look more competent, trustworthy, likable, or attractive.

This study makes several contributions to the literature and live streaming practice. First, unlike previous studies on RPI that were primarily focused on the role of RPI in inducing effort (Hannan et al., 2008, 2013; Tafkov, 2013; Kramer et al., 2016; Berger et al., 2019; Newman et al., 2022), this study explores the informational effect of RPI in the live streaming business, and thus increases our understanding of how RPI influences individuals’ behavior. Second, using a large sample, this study contributes to the live streaming literature by presenting archival evidence of the impacts of RPI in relation to broadcasters on both their behavior and their viewers’ behavior (Wongkitrungrueng et al., 2020; Lin et al., 2021; Liu and Liu, 2021; Chen and Liao, 2022; Guan et al., 2022; Lo et al., 2022; Wu et al., 2022; Xu et al., 2022). Third, the findings of this study contribute to the literature on beauty premiums by presenting a comprehensive framework enabling us to understand how various features of a broadcaster’s appearance (i.e., competence, trustworthiness, likeability, and attractiveness) impact the link between RPI and live streaming performance (Stinebrickner et al., 2019; Póvoa et al., 2020). Finally, from the practical perspective, the findings of this study provide several useful insights for live streaming enterprises and broadcasters.

We have organized the remainder of the paper as follows: In Section II, we review live streaming and RPI research. In Section III, we describe related theories and develop hypotheses. In Section IV, we describe the research design. In Section V, we present the empirical results. In the final section, we draw conclusions.

## Literature review

### Live streaming

The majority of live streaming studies have explored a diverse range of factors that influence viewer behavior, and this body of literature can be divided into two categories based on viewer behavior (Li et al., 2021; Lu et al., 2021; Chen and Liao, 2022; Guan et al., 2022; Liu et al., 2022; Lo et al., 2022; Wu et al., 2022; Xu et al., 2022). The first category focuses on the viewers’ purchase intentions and buying behavior in the live streaming e-commerce context (Lo et al., 2022; Xu et al., 2022). Researchers have established that scarcity persuasion, various experiences, parasocial interaction, price perception, professionalism of the broadcaster, and reciprocal expectations are all factors that influence viewers’ desire to purchase (Lo et al., 2022; Xu et al., 2022), whereas scandals involving the broadcaster could reduce the viewer’s purchase intentions (Xu et al., 2022). The other category focuses on viewers’ tipping behavior (Lee et al., 2019; Lu et al., 2021; Guan et al., 2022; Liu et al., 2022; Wu et al., 2022). A survey by Lee et al. (2019) showed that interaction and content were the two most important factors influencing the viewers’ decision to tip. The authors further suggested that viewers’ anticipation that broadcasters actively participate in social media interactions with them in exchange for tips (i.e., reciprocity) and the desire to stand out from the crowd by tipping (i.e., social image) are their primary motivations for interactions during live streams. A survey conducted by iiMedia Research (2019) also highlighted the importance of social image concerns in relation to viewers’ tips. The survey also identified four other reasons for tipping: content uniqueness, content relevance, broadcaster charisma, and social norms. Similarly, in a randomized controlled field experiment, Lu et al. (2021) identified a positive relationship between audience size and average tip per viewer, suggesting that social image concerns outweigh the need for reciprocity when the audience size increases. Some studies have also explored the mechanisms through which various antecedents affect viewers’ desire to tip and found that viewers’ materialism, value perceptions in relation to the giving quantity, perceived closeness to the broadcaster, and sense of belonging to the viewing crowd influence their tipping behavior (Guan et al., 2022; Liu et al., 2022; Wu et al., 2022). In addition to these studies focusing on viewers’ purchasing and tipping behavior, other studies have investigated the antecedents of users’ “stickiness” and viewing behavior (Li et al., 2021; Chen and Liao, 2022).

Given that the broadcaster is the primary component of a live stream, recent studies have placed increasing emphasis on examining broadcasters’ traits and behavior. For example, Lin et al. (2021) suggested that a happier broadcaster makes the viewer happier and encourages increased viewer participation, particularly in terms of tipping behavior, and that the broadcaster smiles more in return for greater viewer engagement. Furthermore, they found that these effects were more pronounced among broadcasters who had more experience, received more tips, or were more popular in previous live streams. Liu and Liu (2021) indicated that an excessively high revenue-sharing rate established by the platform lowered the broadcaster’s motivation to sell products and investment in the platform’s recommendations. Consequently, both the platform’s and broadcaster’s revenues might be negatively affected by an extremely high revenue-sharing rate. Wongkitrungrueng et al. (2020) identified and summarized four sales approaches and associated combined strategies that broadcasters can use to attract and retain customers in the live streaming context. Xu et al. (2022) found a positive association between broadcaster credibility and brand attitude, while identifying parasocial interactions and broadcaster loyalty as two elements mediating the influence of broadcaster credibility on brand attitude.

### Relative performance information

The positive relationship between RPI and effort, as well as resulting performance, has been widely validated (Hannan et al., 2008, 2013; Tafkov, 2013; Kramer et al., 2016). Two theoretical perspectives, including agency theory and social comparison theory, have been used to explain how RPI affects individual effort and performance (Mahlendorf et al., 2014). Agency theory is often applied to situations in which superiors evaluate subordinates based on RPI and link the outcomes of their evaluations to the subordinates’ compensation contracts. The rationale for using RPI in the evaluation process is that relative performance evaluation can help to mitigate uncertainties (Holmstrom, 1982; Frederickson, 1992). Specifically, because an employee’s performance is a function of effort, abilities, and uncertainties, using RPI to evaluate and compare the performance of employees enables employers to reduce performance measure noise by filtering out common uncertainties (Mahlendorf et al., 2014). Hence, compensating employees based on their relative performance enhances risk-sharing between the employer and the employee, thereby providing an economic incentive for the employee to exert more effort and improve their subsequent performance.

A growing number of experimental studies have examined the role of psychological incentives in RPI (Hannan et al., 2013, 2019; Tafkov, 2013; Newman et al., 2022). This line of research adopts the perspective of social comparison theory and seeks to isolate the behavioral impacts of RPI in the absence of performance incentives, thereby indirectly examining the central tenet of agency theory. Social comparison theory suggests that people aspire to outperform their competitors (Festinger, 1954; Frederickson, 1992). Thus, RPI that reveals a person’s talents and abilities compared with those of others motivates greater effort to surpass those with whom one is compared. In summary, the psychological incentive of RPI involves a social comparison process in which people are focused on the defined objective of RPI by comparing their performance with that of their competitors, evoking a psychological incentive to invest more effort (Festinger, 1954; Locke and Latham, 1990, 2002; Hannan et al., 2013).

Researchers have applied agency theory and social comparison theory to various settings and obtained some insightful outcomes. Hannan et al. (2008) showed that when incentive-based compensation was present, RPI enhanced performance regardless of the accuracy of RPI. However, performance suffered under tournament incentives when RPI was more detailed. Tafkov (2013) found that in an environment where compensation is independent of peer performance, RPI increased performance more among participants who received performance-based contracts than among those who receive flat-wage contracts, with the effect being greater when RPI was publicly available. Newman et al. (2022) suggested that RPI improves individuals’ propensity to explore alternative approaches and assists them in determining whether their exploration will likely benefit or damage their performance.

In addition to the advantages of RPI that have been established in the literature, some studies have identified possible drawbacks to RPI. Hannan et al. (2013) and Hannan et al. (2019) suggested that RPI could distort effort allocation in a multi-task environment. Berger et al. (2019) found that RPI increased both active and passive counterproductive knowledge-sharing behavior, with passive counterproductive behavior occurring more frequently when participants received a performance-based incentive and active counterproductive behavior occurring more frequently when participants received a flat wage. Holderness et al. (2020) found that while low-frequency assigned RPI enhanced performance more than no RPI, high-frequency assigned RPI diminished performance.

## Hypothesis development

### The effects of RPI on live streaming performance

We expect that RPI will improve live streaming performance through two different mechanisms: an effort-eliciting effect and an informational effect.

#### Effort-eliciting effect

Insofar as RPI is important in determining a broadcaster’s income in the live streaming market, the income a broadcaster receives can be regarded as a specific type of performance-based contract (Guan et al., 2022). Because marketing research suggests that the total amount that consumers are prepared to pay for a product during a specific period is relatively steady in a given market (Putsis, 1998; Mittal and Sethi, 2011), the relative stability in terms of the amount viewers are prepared to tip broadcasters on a live streaming platform places the broadcasters in a similar scenario to a zero-sum game. Consequently, the live streaming market is analogous to a dynamic tournament environment in which broadcasters compete to provide viewer-satisfying content in exchange for viewer tips. On the basis of economic theory, which suggests that RPI can significantly increase subsequent performance when an individual’s compensation contract is associated with competitors’ performance (Holmstrom, 1982; Hannan et al., 2008), we expect that RPI in the live streaming market will also increase a broadcaster’s effort, and thus their resulting live streaming performance.

In addition to providing an economic incentive, RPI in the live streaming business can facilitate social comparison (i.e., a psychological incentive; Hannan et al., 2013; Tafkov, 2013). Live streaming performance is a function of a broadcaster’s ability, effort, and common uncertainty, and RPI filters out a significant proportion of the common uncertainty (Mahlendorf et al., 2014), enabling it to communicate more hybrid information pertaining to the broadcaster’s ability and effort. Social comparison theory posits that one of the primary reasons people engage in social comparison is to evaluate their own abilities and observe the difference between their own ability and that of others (Suls and Wheeler, 2000; Brown et al., 2007; Gerber et al., 2018; Zell and Strickhouser, 2020). After obtaining information regarding the performance gap between themselves and their peers from publicly available RPI, broadcasters will attempt to improve their performance ranking by exerting greater effort. Because ability is a relatively constant personal characteristic (Smith and Arnkelsson, 2000), disparities in ability are commonly believed to be more difficult to erase than differences in the level of effort, resulting in the perception that ability has a greater influence on self-image (Smith, 2000; Smith et al., 2002; Zell et al., 2020). Publicly available RPI promotes social comparison and motivates broadcasters to increase their level of effort in an attempt to improve their performance, thereby alleviating image concerns resulting from insufficient ability (Zell et al., 2020).

In accordance with Bonner and Sprinkle’s (2002) framework, which suggested that an employee’s effort will positively affect their task performance, we expect that a broadcaster’s level of effort that is enhanced by RPI will also have a significant positive impact on their live streaming performance (Wongkitrungrueng et al., 2020). In summary, on the basis of both economic and social comparison perspectives, we expect that RPI in the live streaming market can produce an effort-eliciting effect that improves live streaming performance by increasing the broadcaster’s level of effort.

We thus propose the following hypothesis:

*H1*: RPI improves live streaming performance by increasing the broadcaster’s effort. That is, broadcaster effort mediates the relationship between RPI and live streaming performance.

#### Informational effect

Although RPI might encourage broadcasters to exert more effort, we predict that it will also inform viewers regarding the crowd’s preferences, which could lead to herding behavior. The herding effect refers to the propensity of people to imitate the behavior of others (Banerjee, 1992). The occurrence of herding requires both the existence of uncertainty (e.g., informational asymmetry) and the opportunity to observe the behavior of other people and mimic that behavior (Cipriani and Guarino, 2014; Lee et al., 2015; Zhang et al., 2015). When these conditions are met, either an information cascade or herding may arise, which is characterized by people imitating the acts of others while disregarding their own private information (Bikhchandani et al., 1992, 1998; Tucker and Zhang, 2011; Zhang and Liu, 2012; Ding and Li, 2019).

On the basis of insights from the herd behavior literature, we argue that the characteristics of the live streaming environment facilitate the occurrence of herding for the following reasons (Lu et al., 2021). First, there is a considerable variety of broadcasters on the platforms, with significant differences in terms of style and ability. Meanwhile, viewers often lack sufficient time to become acquainted with the broadcasters prior to choosing which broadcaster they will follow. These satisfy the first prerequisite for the herding effect, namely, the existence of informational asymmetry and uncertainty between viewers and broadcasters. Second, the publicly accessible nature of RPI in the live streaming market allows viewers to observe and imitate the viewing crowd’s decisions, thereby satisfying the second prerequisite for the herding effect.

In summary, when a new viewer is uninformed regarding broadcasters, he/she is likely to adapt his/her preferences to match the decisions embedded in RPI regarding the viewing crowd’s choices. In this case, RPI can serve as a signal of the broadcaster’s ability and encourage viewers to make decisions that depend more on the opinions of the crowd and less on their own private information such as their own preferences (Tucker and Zhang, 2011; Zhang and Liu, 2012). Consequently, the greater the RPI regarding a broadcaster, the higher the probability of attracting more viewers and receiving more tips.

We thus propose the following hypothesis:

*H2*: RPI improves live streaming performance by increasing viewer participation. That is, viewer participation mediates the relationship between RPI and live streaming performance.

### The moderating role of broadcaster appearance

Insights from the beauty premium literature, which suggests that physical attractiveness enhances the likelihood that a person will enjoy measurable social and economic advantages (Póvoa et al., 2020), are useful for understanding the moderating role of broadcaster appearance. Research on the beauty premium can be traced back to the 1990s. Hamermesh and Biddle (1994) revealed that employees in the United States with above-average beauty earned 10–15% more than those with below-average beauty, which they termed the “beauty premium.” Since then, the beauty premium has been confirmed in various fields, such as education (Hamermesh and Parker, 2005), CEO selection (Graham et al., 2017), professional sports (Berri et al., 2011), and elections (Berggren et al., 2010), with studies proposing two explanations for its occurrence. The first is based on evolutionary theory, and emphasizes natural selection. Evolutionary theory states that we desire beauty because it delivers a signal regarding fertility-and/or survival-related attributes such as health, athleticism, and intelligence (Langlois et al., 2000; Mobius and Rosenblat, 2006). The second explanation is based on a deeper intuitive relationship between physical attractiveness and positive judgment (Hamermesh and Biddle, 1994; Mulford et al., 1998; Newman and Bloom, 2012). This perspective suggests that humans naturally prefer beauty independent of fitness or health, and that the genetic preference for beauty is instinctive and emotional, arising as a byproduct of information processing in the human brain.

The beauty premium varies across job or task types, and has been found to be particularly evident in jobs that require interpersonal interaction (Mobius and Rosenblat, 2006; Stinebrickner et al., 2019). Because extensive interactions occur between the broadcaster and viewers during live streams, we expect that the beauty premium will be pronounced in the live streaming market, and thus posit that the broadcaster’s beauty will enhance the positive effects of RPI on their live streaming performance. Drawing on these insights suggests that humans value beauty, and thus viewers of live streams hosted by attractive broadcasters are likely to feel more satisfied than those watching live streams hosted by less-attractive broadcasters (Mobius and Rosenblat, 2006). Some neuroscience studies have suggested that visually appealing stimuli trigger the brain’s reward centers, elicit strong positive emotions, and provide enjoyable subjective experiences (Aharon et al., 2001; Leder et al., 2004; Reber et al., 2004; Reimann et al., 2010; Townsend and Shu, 2010), all of which increase the likelihood that viewers will tip during live streams. In summary, viewers might subjectively overestimate the level of effort exerted by attractive broadcasters while underestimating the level of effort exerted by less-attractive broadcasters (Mobius and Rosenblat, 2006). As a result, viewers send more virtual gifts (tips) to attractive broadcasters during live streams, despite the fact that both types of broadcasters exert the same amount of effort. In addition to the fact that physical attractiveness (i.e., beauty) can result in greater economic benefits, recent studies have suggested that other dimensions of appearance, such as competence, trustworthiness, or likability might also attract a premium (Graham et al., 2017). Therefore, to provide a complete framework of how a broadcaster’s appearance affects the effort-eliciting effect of RPI, we examined the moderating role played by the different dimensions of a broadcaster’s appearance. Thus, we propose the following hypotheses:

*H3a*: The indirect relationship between RPI and live streaming performance via broadcaster effort is moderated by the appearance of competence of a broadcaster, such that the effort-eliciting effect of RPI will be greater when broadcasters appear more competent.

*H3b*: The indirect relationship between RPI and live streaming performance via broadcaster effort is moderated by the appearance of trustworthiness of a broadcaster, such that the effort-eliciting effect of RPI will be greater when broadcasters appear more trustworthy.

*H3c*: The indirect relationship between RPI and live streaming performance via broadcaster effort is moderated by the appearance of likability of a broadcaster, such that the effort-eliciting effect of RPI will be greater when broadcasters appear more likeable.

*H3d*: The indirect relationship between RPI and live streaming performance via broadcaster effort is moderated by the appearance of attractiveness of a broadcaster, such that the effort-eliciting effect of RPI will be greater when broadcasters appear more attractive.

To the extent that attractive appearance is seen as an indication of an individual’s ability, an attractive broadcaster will affect viewers’ subjective evaluations of the broadcaster’s competence (Mobius and Rosenblat, 2006). In addition, recent studies have established that attractive individuals have a greater degree of confidence and superior social and communication skills than less-attractive individuals. Similarly, in the live streaming context, attractive broadcasters are likely to possess better social skills, which enables them to increase their interaction with viewers who are drawn to their live streams by RPI (Mobius and Rosenblat, 2006; Peng et al., 2020b), thereby increasing viewer tips. By contrast, less-attractive broadcasters suffer from “lookism” (Peng et al., 2020a). A popular but unattractive broadcaster may have qualities and skills that take longer for the viewers to recognize. New viewers who are unfamiliar with a broadcaster are negatively impacted by the broadcaster’s unattractive appearance, reducing their desire to continue watching the live stream. That is, because of appearance-based discrimination, a viewer who was attracted to the live stream by RPI is less likely to spend time familiarizing themselves with an unattractive broadcaster, thereby decreasing their level of engagement and likelihood of tipping. The above theory can also be used to analyze the impact of other aspects of a broadcaster’s appearance (i.e., competence, trustworthiness, and likeability) on the link between viewer participation and live streaming performance.

We thus propose the following hypotheses:

*H4a*: The indirect relationship between RPI and live streaming performance via viewer participation is moderated by the appearance of competence of a broadcaster, such that the informational effect of RPI is greater when broadcasters appear more competent.

*H4b*: The indirect relationship between RPI and live streaming performance via viewer participation is moderated by the appearance of trustworthiness of a broadcaster, such that the informational effect of RPI is greater when broadcasters appear more trustworthy.

*H4c*: The indirect relationship between RPI and live streaming performance via viewer participation is moderated by the appearance of likeability of a broadcaster, such that the informational effect of RPI is greater when broadcasters appear more likeable.

*H4d*: The indirect relationship between RPI and live streaming performance via viewer participation is moderated by the appearance of attractiveness of a broadcaster, such that the informational effect of RPI is greater when broadcasters appear more attractive.

**Figure 1:**
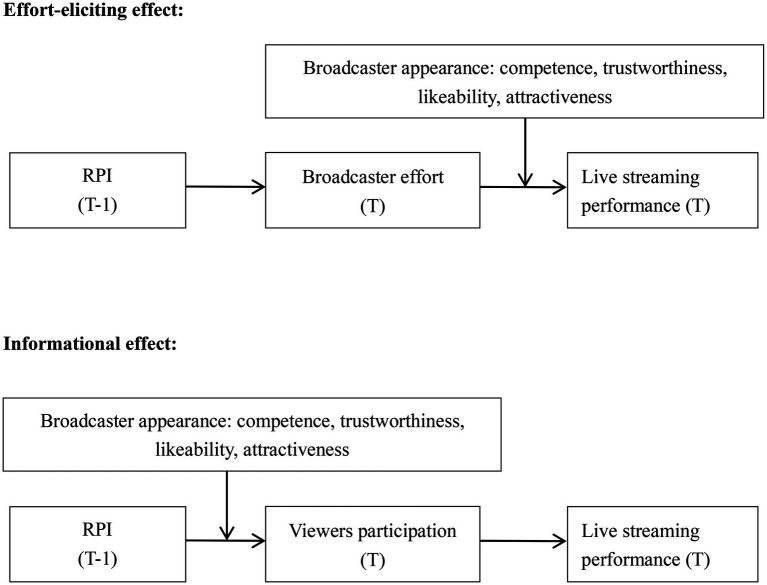
Conceptual framework.

The conceptual framework based on our theory is shown in Figure 1.

## Research design

### Research sample and procedure

We obtained live streaming data from YY Live and Toubang.tv (hereafter YY and Toubang, respectively). YY, a subsidiary of JOYY, is an online live streaming entertainment provider that offers a variety of content including singing, dancing, talk shows, gaming, and other live streaming programs. As one of the most popular live streaming platforms in China, YY reported that the average number of monthly active mobile users of YY reached 274.7 million in the first quarter of 2022.^1^ Toubang is a third-party big data platform focused on the live streaming industry that provides and analyzes live streaming data from mainstream live streaming platforms. As a query and data analysis platform, Toubang automatically records information published by live streaming platforms and builds analysis models based on broadcasters, multi-channel networks, and viewer behavior using real-time live streaming data. In doing so, Toubang ensures that all parties involved in the live streaming industry can acquire and analyze live streaming data.

Our data included 42,166 live streams by 293 broadcasters between 1 May 2020 and 31 October 2020. We obtained our study sample and data in two steps. First, we selected the broadcasters. To obtain an adequate sample size, two members of the research team were required to randomly choose broadcasters by opening YY each night between 8 pm and 10 pm for seven consecutive nights prior to 1 May 2020.^2^ We restricted the broadcaster categories to singing, dancing, and talk shows. The strategy of randomly selecting broadcasters might to some extent reduce the self-selection bias. A total of 327 broadcasters were selected. Second, we matched the selected broadcasters with their live streams, using data obtained from Toubang. After deleting 34 broadcasters with incomplete data, we obtained a final sample of 293 broadcasters and data for 42,166 live streams.^3^ Our final sample included 112 broadcasters of singing shows, 78 broadcasters of dancing shows, and 103 broadcasters of talk shows. The live streaming data included information such as viewer tips, broadcaster popularity, live streaming duration, and viewer comments.

### Definitions of variables

#### Live streaming performance

During the live stream, viewers interact with the broadcaster by commenting, tipping, and sending “likes.” Among the various viewer behaviors, tipping is the most obvious way for viewers to express their appreciation of the broadcaster. Tip income provides significant revenue for live streaming firms, and is also a primary source of revenue for broadcasters. Chen and Xiong (2019) noted that the primary live streaming business model is based on viewers (fans) purchasing gifts for their preferred broadcasters. Therefore, we used the total amount of tips a broadcaster received on a given day as a measure of their performance.

#### Relative performance information

YY and Toubang have created a list that discloses how much broadcasters receive in tips and ranks them based on the amount of tips they receive. Although this list provides rank-score RPI, we constructed actual-score RPI by dividing the total amount of tips that a broadcaster received on a given day by the average value of tips received by all broadcasters in the same category. Hannan et al. (2019) concluded that actual-score RPI provides more detailed information than rank-score RPI. While rank-score RPI allows broadcasters to know their performance ranking relative to their daily broadcasting competitors, it does not provide them with detailed information about the performance differences underlying the rankings. In contrast, actual-score RPI allows broadcasters to discern both their relative performance ranking and performance gap. In the context of live streaming, actual-score RPI increases broadcasters’ knowledge of how they rate compared with their competitors, thereby not only pushing them to work harder in the future, but also alerting them to how much additional effort they should expend. Therefore, actual-score RPI (i.e., the amount of tips received compared with the average amount received by all broadcasters in the category) is an appropriate and informative choice that can be used as a proxy for RPI in the live streaming context.

#### Broadcaster effort

To measure broadcaster effort, we used the total streaming minutes of a broadcaster on a given day as a proxy. Bonner and Sprinkle (2002) divided effort into four dimensions: direction, duration, intensity, and strategic development.^4^ Direction refers to the activity or endeavor in which a person chooses to participate (i.e., what an individual does). The amount of time over which an individual commits cognitive and physical resources to a specific task or activity is referred to as effort duration (i.e., how long a person works). Intensity refers to the degree of attention a person devotes to a job or activity over a period of time (i.e., how hard a person works). Given that live streaming duration can, to some extent, be regarded as a composite of effort duration, direction, and intensity, we measured broadcaster effort in terms of how many minutes a broadcaster live streamed on a given day.

#### Viewer participation

We measured viewer participation by counting the number of viewers who sent real-time comments to the broadcaster during live streams on a given day. Multiple comments from a viewer during the live streams hosted by a broadcaster were treated as a single comment. The live streaming business is inherently real-time. Viewers can connect with the broadcaster *via* texts, allowing a broadcaster to better understand the viewers’ requirements. Regardless of whether viewers send favorable or critical comments to the broadcaster, commenting behavior signifies that the viewer has entered the showroom and is following the broadcaster.

#### Broadcaster appearance

Graham et al. (2017) categorized appearance into four dimensions, competent, trustworthy, likeable, and attractive, and examined the relationship between those dimensions and CEO selection and compensation. We used Graham et al.’s (2017) approach to measure broadcaster appearance using the subjective assessments of independent raters. We recruited six Gen Z raters (two females and four males) who frequently watched live streams to rate the appearance of the broadcasters. Statistics published by QuestMobile showed that Gen Z accounts for the majority of live stream viewers in China (QuestMobile, 2019). Furthermore, YY has more than 11.77 million monthly Gen Z users, ranking it number one in the pan-entertainment live streaming sector.^5^

We delivered the questionnaires to the six raters through Wenjuanxing, a professional online questionnaire platform in China. The questionnaires included photographs of selected broadcasters and related questions asking them to evaluate the broadcasters’ appearance in four dimensions. We developed the questionnaires using the following steps. First, we looked for suitable photographs of sample broadcasters on the YY website. The photographs we chose had to satisfy several criteria: clear facial features, a natural expression, high resolution, and a conventional pose. Second, the 293 broadcasters who we selected were divided into three groups based on content type to enable the raters to compare broadcasters who generated similar content. Hence, every rater received three questionnaires. Raters had 2 days to complete the questionnaires by scoring the four dimensions of each broadcaster’s appearance – competence, trustworthiness, likability, and attractiveness – using a five-point Likert-type scale ranging from 1 (low) to 5 (high). We then summed the rater’s scores for each appearance dimension to construct the relevant appearance variables.

**Table:** 

Not competent → very competent				
1	2	3	4	5
Not trustworthy → very trustworthy				
1	2	3	4	5
Not likeable → very likeable				
1	2	3	4	5
Not attractive → very attractive				
1	2	3	4	5

## Empirical results

### Descriptive statistics

Table 1 presents the descriptive statistics and correlations for the variables of interest. It can be seen from Table 1 that RPI is significantly positively correlated with broadcaster effort (*r* = 0.087, *p* < 0.01), viewer participation (*r* = 0.390, *p* < 0.01), and live streaming performance (*r* = 0.5, *p* < 0.01), broadcaster effort is positively correlated with live streaming performance (*r* = 0.138, *p* < 0.01), and viewer participation is also significantly positively correlated with live streaming performance (*r* = 0.432, *p* < 0.01). These preliminary results provided a basis for further examination of our predictions.

**Table 1:** Descriptive statistics and correlations.

	N	Min	Max	Mean	SD	1	2	3	4	5	6	7	8
1. RPI	42,166	0	44.93	1.024	1.821	1	0.087***	0.390***	0.104***	0.096***	0.098***	0.143***	0.500***
2. Broadcaster effort	42,166	1803	86,399	22554.580	9533.317	0.087***	1	0.140***	−0.103***	−0.130***	−0.100***	−0.072***	0.138***
3. Viewers participation	42,166	0	5,155	125.66	244.699	0.390***	0.140***	1	0.128***	0.085***	0.083***	0.036***	0.432***
4. Competence	42,166	11	26	18.6	2.723	0.104***	−0.103***	0.128***	1	0.728***	0.627***	0.640***	0.104***
5. Trustworthiness	42,166	10	26	17.49	2.664	0.096***	−0.130***	0.085***	0.728***	1	0.805***	0.711***	0.100***
6. Likeability	42,166	10	26	18.02	3.036	0.098***	−0.100***	0.083***	0.627***	0.805***	1	0.756***	0.103***
7. Attractiveness	42,166	8	26	16.48	3.65	0.143***	−0.072***	0.036***	0.640***	0.711***	0.756***	1	0.137***
8. Performance	42,166	0	58,081,954	490063.32	1034551.574	0.500***	0.138***	0.432***	0.104***	0.100***	0.103***	0.137***	1

*, **, and *** denote two-tailed significance at the 10, 5, and 1% levels, respectively. Broadcaster effort is measured by how many minutes a broadcaster streamed on a given day. Viewer participation is measured by the number of viewers who sent real-time comments to the broadcaster during live streams on a given day. Broadcaster appearance (competence, trustworthiness, likeability, and attractiveness) was scored by six raters. YY use the “Reli” as their unit for viewer tips for live streaming performances.

### Hypothesis testing

#### Effects of RPI on live streaming performance

H1 states that RPI improves live streaming performance by increasing broadcaster effort (i.e., the mediating effect of broadcaster effort), while H2 states that RPI improves live streaming performance by increasing viewer participation (i.e., the mediating effect of viewer participation). In recent years, many scholars have questioned the statistical validity of the hierarchical regression method (Yan et al., 2021). Thus, to test our hypotheses, we performed bootstrapping using the PROCESS SPSS Macro (model 4) created by Hayes (2018). This software is used to investigate the impact of one or more mediating or moderating factors on the relationship between the independent and dependent variables. In addition, the number of bootstrap samples was set to 5,000 and the confidence interval (CI) was set to 95% to test our hypotheses. The results are presented in Tables 2, 3. It can be seen from Tables 2, 3 that RPI had a significant positive effect on live streaming performance (total effect = 283987.732, 95% CI = 279291.168, 288684.296).

**Table 2:** Mediating effect of broadcaster effort.

Panel A: Mediation 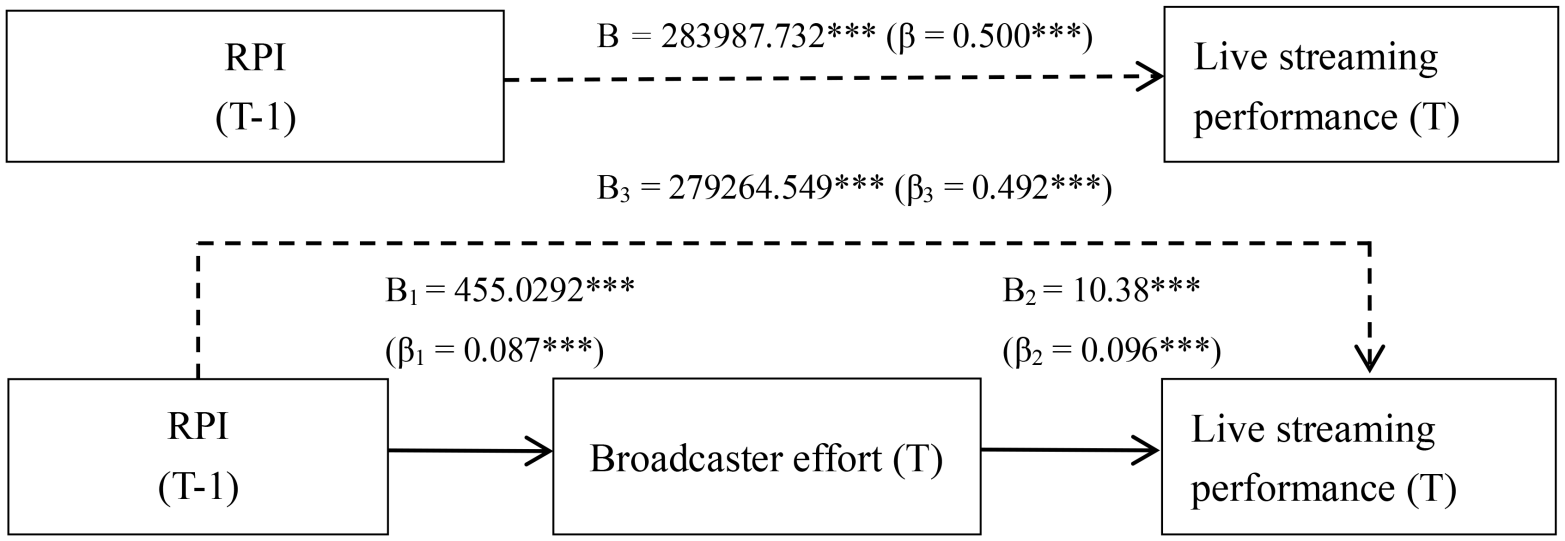				
**Panel B: Bootstrap Results**				
	**Effect**	**SE**	**LLCI**	**ULCI**
Indirect effect	4723.183	375.585	4010.022	5495.346
Direct effect	279264.549	12076.887	256467.279	304040.215
Total effects	283987.732	2396.1814	279291.168	288684.296

*, **, and *** denote two-tailed significance at the 10, 5, and 1% levels, respectively. “B” and “β” indicate non-standardized and standardized values, respectively. The numbers on the arrows represent the estimated coefficients based on the following system of equations:

Live streaming performance = β × RPI + ε_1_ Broadcaster effort = β_1_ × RPI + ε_2_ Live streaming performance = β_2_ × broadcaster effort + β_3_ *×* RPI + ε_3_

**Table 3:** Mediating effect of viewer participation.

Panel A: Mediation Model 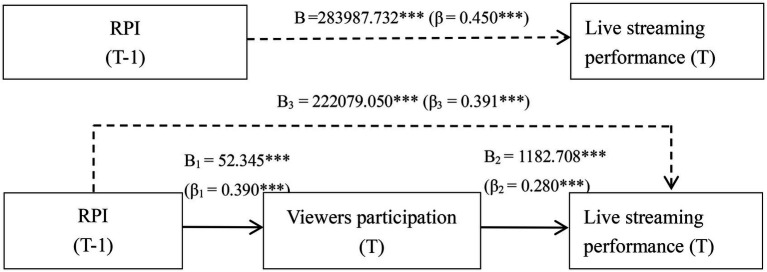				
**Panel B: Bootstrap Results**				
	**Effect**	**SE**	**LLCI**	**ULCI**
Indirect effect	61908.682	5604.561	51817.366	73825.172
Direct effect	222079.050	11089.995	200870.266	244142.376
Total effects	283987.732	2396.181	279291.168	288684.296

*, **, and *** denote two-tailed significance at the 10, 5, and 1% levels, respectively. “B” and “β” indicate non-standardized and standardized values, respectively. The numbers on the arrows represent the estimated coefficients based on the following system of equations:

Live streaming performance = β × RPI + ε_1_ Broadcaster effort = β_1_ × RPI + ε_2_ Live streaming performance = β_2_ × broadcaster effort + β_3_ *×* RPI + ε_3_

##### The mediating effect of broadcaster effort (effort-eliciting effect)

We found that RPI significantly increased broadcaster effort (B_1_ = 455.029, *t* = 17.916, *p* < 0.01), which in turn improved live streaming performance (B_2_ = 10.380, *t* = 22.729, *p <* 0.01, see Table 2). As can be seen from Table 2, both the indirect effect of RPI on live streaming performance through increasing broadcaster effort (indirect effect = 4723.183, 95% CI = 4010.022, 5495.346) and the direct effect of RPI on live streaming performance were significant (direct effect = 279264.549, 95% CI = 256467.279, 304040.215). Thus, H1 is supported.

##### The mediating effect of viewer participation (informational effect)

As can be seen from Table 3, RPI significantly increased viewer participation (B_1_ = 52.345, *t* = 86.851, *p <* 0. 01), which in turn improved live streaming performance (B_2_ = 1182.708, *t* = 63.980, *p <* 0.01). The results presented in Table 3 show that the indirect effect of viewer participation on live streaming performance (indirect effect = 61908.682, 95% CI = 51817.366, 73825.172) was statistically significant. Thus, H2 is supported.

#### Moderating role of broadcaster appearance

##### Effort-eliciting effect

H3a–H3d state that the second half of the mediated model (mediated by broadcaster effort) was moderated by the broadcaster’s appearance, and thus model 14 in the PROCESS SPSS Macro was used to examine the moderated mediation model. The number of bootstrap samples was set to 5,000 and the CI was set to 95% to test the moderated mediation hypotheses. We conducted four moderated mediation tests using the broadcaster’s appearance of competence, trustworthiness, likability, and attractiveness as moderators. The results are presented in Table 4. It can be seen from Table 4 that all dimensions were positively correlated with live streaming performance, and the coefficient of the interaction term between effort and appearance dimension was significant in all models. Specifically, the coefficient of the interaction term was 1.88 (*p* < 0.01) when moderated by competence, 1.55 (*p* < 0.01) when moderated by trustworthiness, 1.25 (*p* < 0.01) when moderated by likeability, and 1.45 (*p* < 0.01) when moderated by attractiveness (see Panels A–D of Table 4). Thus, H3a–H3d are supported.

**Table 4:** Moderating effects of broadcaster appearance when mediated by broadcaster effort.

Factors	Broadcaster effort					Live streaming performance				
	Β	β	se	t	p	Β	β	se	t	p
**Panel A: Moderating effect of competent look**										
RPI	455.03	0.087	25.40	17.92	< 0.01	273729.77	0.482	2399.80	114.06	< 0.01
Effort						11.41	0.105	0.4583	24.90	< 0.01
Competence						25528.07	0.067	1609.78	15.86	< 0.01
Effort × competence						1.88	0.047	0.17	11.19	< 0.01
R^2^	0.0076					0.27				
F	320.98					3802.46				
**Panel B: Moderating effect of trustworthy look**										
RPI	455.03	0.087	25.40	17.92	< 0.01	274649.74	0.484	2396.09	114.62	< 0.01
Effort						11.74	0.108	0.46	25.49	< 0.01
Trustworthiness						27286.33	0.070	1651.10	16.53	< 0.01
Effort × trustworthiness						1.55	0.038	0.17	9.31	< 0.01
R^2^	0.0076					0.26				
F	320.98					3797.04				
**Panel C: Moderating effect of likeable look**										
RPI	455.03	0.087	25.40	17.92	< 0.01	274823.11	0.484	2396.03	114.70	< 0.01
Effort						11.68	0.108	0.46	25.31	< 0.01
Likeability						23158.17	0.068	1440.43	16.08	< 0.01
Effort × likeability						1.25	0.035	0.14	8.72	< 0.01
R^2^	0.0076					0.27				
F	320.98					3792.01				
**Panel D: Moderating effect of attractive look**										
RPI	455.03	0.087	25.40	17.92	< 0.01	271521.60	0.478	2407.97	112.76	< 0.01
Effort						11.35	0.105	0.46	24.85	< 0.01
Attractiveness						21845.50	0.077	1199.01	18.22	< 0.01
Effort × attractiveness						1.45	0.049	0.13	11.42	< 0.01
R^2^	0.0076					0.27				
F	320.98					3835.09				

To further examine the moderating influence of broadcaster appearance, we separated each appearance dimension into low (mean-1 standard deviation (SD)) and high (mean + 1 SD) groups and conducted simple slope analyses. As can be seen from Table 5, the 95% CI did not contain zero in any model and every appearance dimension affected the relationship between effort and live streaming performance. It can be seen from Figure 3 that broadcaster effort is a stronger predictor of live streaming performance when the broadcaster looks more competent, trustworthy, likable, or attractive.

**Table 5:** Conditional indirect effect of various dimensions of broadcaster appearance when mediated by broadcaster effort.

	Effect	Boot SE	LLCI	ULCI
**Panel A: Conditional effect of competent look**				
Low (Mean − 1 SD)	2857.90	316.83	2267.61	3483.19
Medium	5192.98	405.64	4400.67	6013.33
High (Mean + 1 SD)	7528.05	671.25	6245.22	8894.16
**Panel B: Conditional effect of trustworthy look**				
Low (Mean − 1 SD)	3461.63	389.45	2726.29	4234.58
Medium	5345.42	427.10	4552.01	6240.70
High (Mean + 1 SD)	7229.20	729.60	5952.80	8811.98
**Panel C: Conditional effect of likeable look**				
Low (Mean − 1 SD)	3583.03	344.29	2922.62	4273.20
Medium	5313.98	413.75	4529.38	6153.24
High (Mean + 1 SD)	7044.93	664.27	5807.54	8405.73
**Panel D: Conditional effect of attractive look**				
Low (Mean − 1 SD)	2748.83	293.82	2199.24	3337.22
Medium	5163.52	396.13	4428.93	5955.61
High (Mean + 1 SD)	7578.22	653.45	6342.70	8905.97

**Figure 2:**
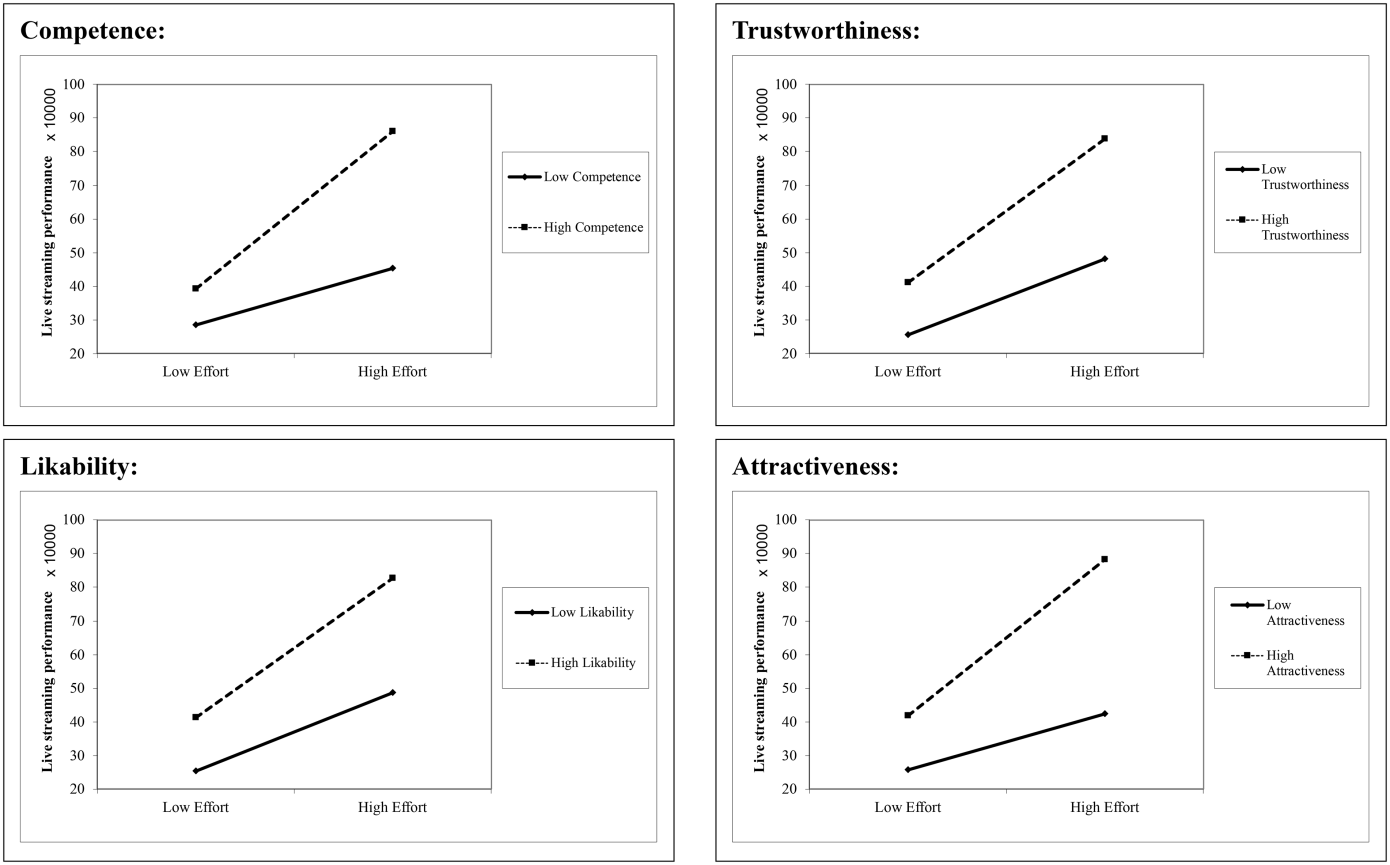
Relationship between broadcaster effort and live streaming performance for high and low levels of each dimension of broadcaster appearance.

##### Informational effect

We used the PROCESS SPSS Macro (model 7) to test H4a–H4d, which state that the informational effect of RPI will be more pronounced when broadcasters appear more competent, trustworthy, likable, or attractive, respectively. Similar to the strategy and parameters described above, we implemented a series of moderated mediation models in which one of the dimensions of broadcaster appearance served as the moderator. The results are presented in Table 6. It can be seen from Table 6 that the coefficient of the interaction term between RPI and appearance dimension was significantly positive in all models (competence: B = 0.73, *p* < 0.01; trustworthiness: B = 0.66, *p* < 0.01; likeability: B = 1.53, *p* < 0.01; attractiveness: B = 1.74, *p* < 0.01). Thus, H4a–H4d are supported.

**Table 6:** Moderating effects of broadcaster appearance when mediated by viewer participation.

Factors	Viewers participation					Live streaming performance				
	Β	β	se	t	p	Β	β	se	t	p
**Panel A: Moderating effect of competent look**										
RPI	50.38	0.375	0.63	79.54	< 0.01	222079.05	0.391	2483.95	89.41	< 0.01
Competence	8.02	0.089	0.40	19.86	< 0.01					
RPI × competence	0.73	0.015	0.20	3.74	< 0.01					
Participation						1182.71	0.280	18.49	63.98	< 0.01
R^2^	0.16					0.32				
F	2672.50					9751.46				
**Panel B: Moderating effect of trustworthy look**										
RPI	50.87	0.379	0.64	79.21	< 0.01	222079.05	0.391	2483.95	89.41	< 0.01
Trustworthiness	4.27	0.047	0.41	10.32	< 0.01					
RPI × trustworthiness	0.66	0.013	0.16	3.99	< 0.01					
Participation						1182.71	0.280	18.49	63.98	< 0.01
R^2^	0.15					0.32				
F	2564.71					9751.46				
**Panel C: Moderating effect of likeable look**										
RPI	49.67	0.370	0.65	76.89	< 0.01	222079.05	0.391	2483.95	89.41	< 0.01
Likeability	3.63	0.045	0.36	10.00	< 0.01					
RPI × likeability	1.53	0.035	0.17	9.08	< 0.01					
Participation						1182.71	0.280	18.49	63.98	< 0.01
R^2^	0.16					0.32				
F	2586.72					9751.46				
**Panel D: Moderating effect of attractive look**										
RPI	49.75	0.370	0.67	74.77	< 0.01	222079.05	0.391	2483.95	89.41	< 0.01
Attractiveness	−1.18	−0.018	0.30	−3.90	< 0.01					
RPI × attractiveness	1.74	0.047	0.16	10.98	< 0.01					
Participation						1182.71	0.280	18.49	63.98	< 0.01
R^2^	0.15					0.32				
F	2568.93					9751.46				

Table 7 presents the results of the simple slope analyses. It can be seen from Table 7 and Figure 4 that RPI has a greater positive impact on viewer participation when broadcasters appear more competent, trustworthy, likeable, or attractive.

**Table 7:** Conditional indirect effect of various dimensions of broadcaster appearance when mediated by viewer participation.

	Effect	Boot SE	LLCI	ULCI
**Panel A: Conditional effect of competent look**				
Low (Mean − 1 SD)	57236.99	5484.13	47320.50	68668.96
Medium	59588.70	5358.44	49831.60	70987.60
High (Mean + 1 SD)	61940.42	5759.57	51644.52	74380.74
**Panel B: Conditional effect of trustworthy look**				
Low (Mean − 1 SD)	58093.99	5496.16	48399.38	70099.81
Medium	60159.17	5368.53	50635.20	71866.74
High (Mean + 1 SD)	62224.35	5689.75	52216.33	74427.48
**Panel C: Conditional effect of likeable look**				
Low (Mean − 1 SD)	53253.37	5204.11	43867.55	64389.86
Medium	58749.37	5224.70	49429.98	69830.74
High (Mean + 1 SD)	64245.38	5882.72	53825.05	76814.56
**Panel D: Conditional effect of attractive look**				
Low (Mean − 1 SD)	51329.57	5173.32	42010.47	62379.38
Medium	58837.81	5319.26	49141.87	70333.90
High (Mean + 1 SD)	66346.06	6213.20	55133.87	79625.11

**Figure 3:**
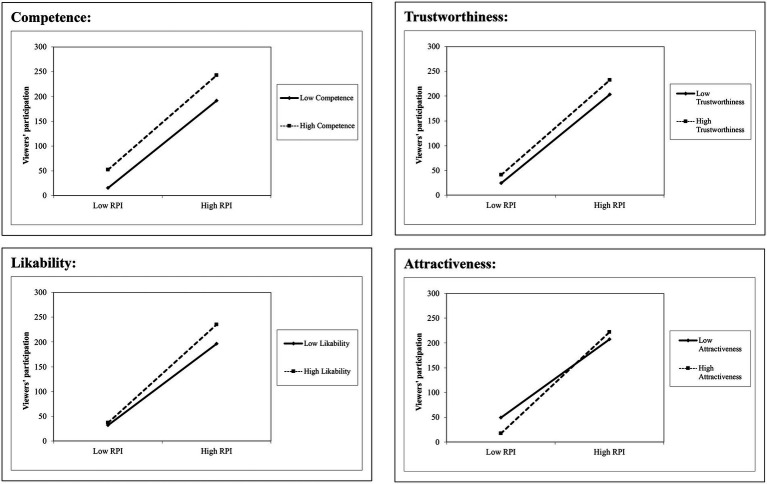
Relationship between RPI and viewer participation for high and low levels of each dimension of broadcaster appearance.

Several conclusions can be drawn from the above results. First, we confirmed the effort-inducing effect of RPI in a live streaming environment. This finding is consistent with those of previous studies in other contexts such as production tasks (Murthy and Schafer, 2011). Second, a novel finding is that new customers (viewers) will be inclined to adopt the crowd-based information embedded in RPI, thereby significantly aligning their choices with those of others. Finally, broadcaster appearance is important in the live-streaming industry. This finding is similar to that of Chen and Liao (2022), but provides additional insight through the inclusion of all four dimensions of broadcaster appearance.

## Discussion

### Theoretical contributions

Our study contributes to the literature in a number of ways. First, it enriches the RPI literature by revealing the effort-eliciting and informational effects of RPI in the live-streaming market. Most previous studies on the influence of RPI on task performance have focused on the function of RPI in inducing effort (Hannan et al., 2008, 2013; Tafkov, 2013; Kramer et al., 2016; Berger et al., 2019; Newman et al., 2022), whereas few studies have evaluated the informational effect of RPI from an external perspective. Given that advances in Internet-based technology and the growth of live streaming businesses have made RPI readily accessible by participants in the live streaming industry, we explored how RPI affects live streaming performance from the perspectives of both broadcasters and viewers. In doing so, we contribute to the RPI literature by not only validating the effort-inducing effect of RPI in a live streaming environment, but also revealing the informational effect of RPI on the decision-making of viewers. The results of our study enhance our understanding of the causal relationship between RPI and subsequent live streaming performance, thereby making a novel contribution to social comparison theory.

The second contribution of our study is that it complements the existing live streaming literature by providing large-sample archival evidence of the behavioral effects of RPI in the live streaming industry. Although live streaming businesses have attracted growing attention from different academic fields in recent years, this stream of research is still in its infancy (Chen and Liao, 2022). Extant live streaming studies have identified the factors influencing viewer behavior (Li et al., 2021; Chen and Liao, 2022; Guan et al., 2022; Liu et al., 2022; Lo et al., 2022; Wu et al., 2022; Xu et al., 2022), and in recent years there has been an increasing focus on the behavior and personal characteristics of broadcasters (Wongkitrungrueng et al., 2020; Lin et al., 2021; Liu and Liu, 2021; Wu et al., 2022; Xu et al., 2022), who are an essential component of live streams. The results of our study elucidate the mechanisms by which broadcasters’ historical performance information affects viewers’ tips, and how broadcaster appearance moderates these mechanisms. Thus, the findings of our study simultaneously add to the body of literature on viewer behavior and that on broadcaster traits.

Finally, the results of this study complement the behavioral labor economics literature by investigating the effects of the beauty premium in relation to the live streaming business (Póvoa et al., 2020). Using survey-based or archival data relating to the labor market, previous studies on the beauty premium have mostly examined whether, and if so how, a particular aspect of a worker’s appearance (i.e., attractiveness) affects their income level (Peng et al., 2020b). We extend this body of literature in two ways. First, unlike previous studies that explored the impact of the beauty premium across several sectors, our study focused on a single sector, namely, the live streaming industry. In addition, the results of our study provide a comprehensive framework for understanding how different aspects of a broadcaster’s appearance influence the relationship between RPI and live streaming performance. More precisely, we identify the moderating role of a broadcaster’s appearance of competence, trustworthiness, likeability, or attractiveness.

### Practical contributions

The findings of this study will be of interest to both broadcasters and live streaming platforms. First, our findings suggest that RPI is conducive to enhancing viewer tips by either increasing the broadcaster’s level of effort or encouraging viewer participation. Given that viewer tips are an important source of revenue for both broadcasters and live streaming platforms, and that broadcasters play a significant role in determining viewer behavior, it is crucial for live streaming platforms to develop an effective performance information sharing system through which broadcasters’ performance information can be made available to both broadcasters and viewers. For instance, the live streaming platform could prominently display the broadcaster performance ratings list on its website or app, or periodically transmit the broadcasters’ performance ratings to participants. However, given that previous studies have identified some potential disadvantages of publicly available RPI when it is provided too frequently or too precisely (Hannan et al., 2008; Holderness et al., 2020), live streaming platforms should consider how to design various elements of RPI in an effort to maximize the benefits of RPI and minimize the disadvantages of making it publicly available.

Second, live streaming companies should develop strategies to amplify the positive effects of RPI. Our findings suggest that RPI has both an effort-eliciting effect and an informational effect. Therefore, the desirable effects of RPI on live streaming performance could be enhanced if live streaming platforms introduced policies aimed at increasing broadcaster effort and/or viewer participation. In terms of strategies aimed at increasing broadcaster effort, live streaming companies could increase the broadcasters’ share of gift revenue, thereby providing broadcasters with a greater incentive to live stream (Liu and Liu, 2021). Introducing talent competitions among broadcasters is another promising strategy to increase broadcaster effort. To amplify the informational effect, platforms should optimize the recommendation system so that it recommends the broadcaster with the most positive RPI to viewers. In addition, live streaming platforms could provide an online forum for each broadcaster to encourage extensive interactions among all participants. The forum posts enable new viewers to learn more about the choices and justifications of other viewers, thereby enhancing viewers’ understanding of the broadcaster’s abilities.

Finally, our findings concerning the moderating effects of the various dimensions of broadcaster appearance reveal that both effort-eliciting and informational effects are more pronounced when the broadcaster is perceived as more competent, trustworthy, likable, or attractive. Therefore, broadcasters should dress formally and wear appropriate makeup in an effort to portray a favorable exterior image, thereby satisfying viewer expectations (Póvoa et al., 2020). Meanwhile, live streaming platforms should introduce policies that specify dress codes for broadcasters. For instance, platforms should prohibit broadcasters from wearing unusual or provocative clothing in an attempt to be eye-catching.

### Limitations and future research

This study has some limitations that should be addressed in future research. First, although the strategy of selecting broadcasters at random reduced the likelihood of self-selection bias to some extent, our findings might still be skewed because a degree of subjectivity related to personal preference was involved in the selection process. In addition, given that endogeneity is not properly accounted for in the study design, the findings might have been influenced by this methodological problem. Future research should use an experimental design that eliminates these issues by strictly randomizing the broadcaster selection process.

The second potential problem relates to the measurement of the live streaming performance. Because gifting is one of the main factors determining the revenue of both broadcasters and platforms, we simply used viewer tips as a proxy for live streaming performance, ignoring all other performance metrics. However, we acknowledge that various non-pecuniary performance measures are also important in the context of live streaming, such as the duration of a viewer’s stay in the showroom and how many “likes” the broadcaster receives. Future research should explore whether, and if so, how non-pecuniary performance information affects the behavior of broadcasters. Related to this, an interesting topic for future research is to examine how non-pecuniary performance information such as “likes,” relative to viewer tips, affects broadcaster’s behavior during live streams. Future research could also examine the joint effects of viewers’ tips and “likes” on broadcaster behavior. In addition, compared to traditional consumption processes, viewer behavior (i.e., tipping, sending “likes,” or comments) during live streams is more observable and accessible, providing an ideal opportunity for future research to examine the relationship between consumers’ cognitive decision-making algorithms and their visual-based choices in live streaming scenarios using machine-learning technology (Andronie et al., 2021; Lin et al., 2021; Hopkins, 2022; Kliestik et al., 2022).

Finally, we restricted the category of broadcasters to the pan-entertainment industry because entertainment accounts for a significant proportion of all live streaming services (Chen and Xiong, 2019). However, the findings of this study based on pan-entertainment live streams might not be applicable to e-commerce settings, because they operate under different modes. Thus, to strengthen the generalizability of our results, future research should examine e-commerce live streaming (Wongkitrungrueng et al., 2020), which is a significant and growing live streaming sector.

## Conclusion

Researchers in various disciplines are devoting considerable attention to the live streaming industry in light of the tremendous development of live streaming businesses. Unlike other industries, the live streaming industry makes RPI regarding broadcasters readily available to all participants, although we know little about the behavioral effects of RPI in this context. Thus, in an attempt to fill this gap, this study examined the effects of broadcasters’ RPI on live streaming performance. Using data from 42,166 live streams by 293 broadcasters and drawing on economic and social comparison theory, as well as insights regarding herd behavior and the beauty premium, we developed and tested models focusing on both the effort-eliciting and informational effects of RPI, as well as the moderating effects of broadcaster appearance. The results of this study indicate that RPI can either increase a broadcaster’s level of effort or encourage viewer participation, thereby improving subsequent live streaming performance, and that these effects are more pronounced when broadcasters appear more competent, trustworthy, likable, or attractive. On the basis of these findings, this study contributes to the RPI and live streaming literature by demonstrating that providing RPI regarding broadcasters can result in effort-eliciting and informational benefits in the live streaming industry. Furthermore, the findings of this study contribute to the behavioral labor economics literature by identifying the effects of the beauty premium in the context of the live streaming industry.

## Data availability statement

The original contributions presented in the study are included in the article/Supplementary material, further inquiries can be directed to the corresponding author.

## Ethics statement

The studies involving human participants were reviewed and approved by Xiamen University. The patients/participants provided their written informed consent to participate in this study.

## Author contributions

YC, XH, and SZ contributed to conception, design of the study, organized the database, and performed the statistical analysis. XH and SZ wrote the first draft of the manuscript. All authors wrote sections of the manuscript. All authors contributed to the article and approved the submitted version.

## Funding

YC would like to acknowledge the financial support received from National Natural Science Foundation of China (Grant# 72172132).

## Conflict of interest

SZ was employed by the company Ernst & Young (China) Hua Ming LLP Hangzhou Branch, Hangzhou, China.

The remaining authors declare that the research was conducted in the absence of any commercial or financial relationships that could be construed as a potential conflict of interest.

## Publisher’s note

All claims expressed in this article are solely those of the authors and do not necessarily represent those of their affiliated organizations, or those of the publisher, the editors and the reviewers. Any product that may be evaluated in this article, or claim that may be made by its manufacturer, is not guaranteed or endorsed by the publisher.

## Footnotes

^1^Seehttps://ir.joyy.com/news-releases/news-release-details/joyy-reports-first-quarter-2022-unaudited-financial-results.

^2^Previous studies have found that both broadcasters and viewers are most engaged during this time slot (Lin et al., 2021).

^3^Live streams of less than 30 min are invalidated by YY. Consequently, our sample excluded 233 invalid live streams, leaving 42,166 live streams.

^4^Strategic development is the long-term effort that influences long-term performance (Bonner and Sprinkle, 2002). We exclude it from the theoretical analysis because it is less likely that broadcasters could develop effective strategies in the short run.

^5^See https://www.joyy.com/post/85827156.
